# Improved B cell development in humanized NOD*‐scid IL2Rγ^null^* mice transgenically expressing human stem cell factor, granulocyte‐macrophage colony‐stimulating factor and interleukin‐3

**DOI:** 10.1002/iid3.124

**Published:** 2016-08-28

**Authors:** Sonal Jangalwe, Leonard D. Shultz, Anuja Mathew, Michael A. Brehm

**Affiliations:** ^1^ Program in Molecular Medicine, Diabetes Center of Excellence™University of Massachusetts Medical SchoolWorcesterMassachusetts01605USA; ^2^ The Jackson LaboratoryBar HarborMaine04609USA; ^3^ Department of Cell and Molecular BiologyUniversity of Rhode IslandProvidenceRhode Island02903USA

**Keywords:** B cells, cytokine, hematopoietic stem cells, humanized mice, SCID

## Abstract

**Introduction:**

Immunodeficient mice engrafted with human immune systems support studies of human hematopoiesis and the immune response to human‐specific pathogens. A significant limitation of these humanized mouse models is, however, a severely restricted ability of human B cells to undergo class switching and produce antigen‐specific IgG after infection or immunization.

**Methods:**

In this study, we have characterized the development and function of human B cells in NOD‐*scid IL2Rγ^null^* (NSG) mice transgenically expressing human stem cell factor (SCF), granulocyte macrophage colony‐stimulating factor (GM‐CSF), and IL‐3 (NSG‐SGM3) following engraftment with human hematopoietic stem cells, autologous fetal liver, and thymic tissues (bone marrow, liver, thymus or BLT model). The NSG‐SGM3 BLT mice engraft rapidly with human immune cells and develop T cells, B cells, and myeloid cells.

**Results:**

A higher proportion of human B cells developing in NSG‐SGM3 BLT mice had a mature/naive phenotype with a corresponding decrease in immature/transitional human B cells as compared to NSG BLT mice. In addition, NSG‐SGM3 BLT mice have higher basal levels of human IgM and IgG as compared with NSG BLT mice. Moreover, dengue virus infection of NSG‐SGM3 BLT mice generated higher levels of antigen‐specific IgM and IgG, a result not observed in NSG BLT mice.

**Conclusions:**

Our studies suggest that NSG‐SGM3 BLT mice show improved human B cell development and permit the generation of antigen‐specific antibody responses to viral infection.

## Introduction

Immunodeficient mice engrafted with functional human immune systems, termed “humanized mice,” are increasingly being utilized to study human immunity as well as infections, autoimmunity, allergies, organ transplantation, vaccine development, and immune regulation 1, 2. A key factor for the successful generation of humanized mice is the use of optimal mouse strains that enable the survival of engrafted human cells and tissues. Immunodeficient *scid*, *Rag1^null^* or *Rag2^null^* mice bearing mutations within the IL2 receptor gamma chain (*IL2rg*) gene support the robust engraftment of human immune cells and tissues and the development of functional human immune systems 3, 4, 5, 6. A number of different strategies have been used to engraft human immune systems in immunodeficient mice. These strategies include injection of human PBL (Hu‐PBL‐SCID model) 7, injection of hematopoietic stem cells (HSC) (Hu‐SRC‐SCID model) 3, 5, and implantation of human fetal liver and thymus tissues in combination with injection of autologous HSC (BLT or bone marrow/thymus/liver model) 8, 9. Each of these humanized mouse models has distinct strengths and weaknesses, and selection of the appropriate model is dependent on the specific experimental question being addressed.

Current efforts to further enhance immune system development and function in humanized mouse models have focused on improving specific human immune cell populations. One major limitation of the existing humanized mouse models is the severely limited ability of human B cells in these mice to undergo class switching and affinity maturation in response to pathogens or immunization with protein antigens 10. Antigen‐specific antibody responses are generated in these mice but they are largely of the IgM isotype with very low IgG titers, which suggests inefficient class switching 11, 12. The restricted B cell responses in humanized mice are an obstacle for studies of vaccine development and infectious diseases where humoral responses predominate. The limited ability of human B cells to undergo class switching in humanized mice is attributed to several factors including impaired T and B cell maturation, lack of secondary lymphoid structures in the peripheral lymphoid organs, poor reconstitution of myeloid antigen‐presenting cells (APCs), and insufficiency of human cytokines 13, 14, 15, 16, 17. Examples of approaches to improve human B cell function include expression of HLA class II and expression or injection of human cytokines that facilitate hematopoiesis and lymphocyte differentiation. While these approaches have enabled improvements in B cell function, generation of high levels of antigen‐specific IgG remains problematic.

In this study, we evaluated human B cell development and function in humanized NSG mice constitutively expressing human stem cell factor (SCF), granulocyte‐macrophage colony‐stimulating factor (GM‐CSF), and interleukin‐3 (IL‐3), also known as NSG‐SGM3 mice. Previous studies have shown that NSG‐SGM3 mice have significantly improved engraftment of human acute myeloid leukemia (AML) cells as well as long‐term pre‐leukemic myeloid cell cultures 18. Moreover, NSG‐SGM3 mice engrafted with human CD34^+^ HSC have elevated levels of neutrophils 19 and other granulocytes 20, myeloid dendritic cells (mDCs) as well as CD4^+^ cells with a lineage skewing toward regulatory T cells (Tregs) that were functionally and phenotypically equivalent to human Tregs 21. To test the ability of NSG‐SGM3 mice to support human B cell development and function, these mice along with NSG mice were transplanted with human fetal thymic and liver tissues and autologous fetal liver derived CD34^+^ HSC to generate BLT mice. Our results show that NSG‐SGM3 BLT mice have enhanced human B cell development, with higher levels of mature naïve B cells and lower levels of immature transitional and transitional B cells as compared with NSG BLT mice. NSG‐SGM3 BLT mice also had higher basal levels of human IgM and IgG in the plasma as compared with control NSG BLT mice. Finally, infection of NSG‐SGM3 BLT mice with dengue virus stimulated the generation of antigen‐specific IgM and IgG responses at levels higher than NSG BLT mice. Our results indicate that NSG‐SGM3 mice support enhanced development and maturation of human B cells and will be a useful model to study human antigen‐specific B cell responses.

## Materials and Methods

### Mice

NOD.*Cg‐Prkdc^scid^Il2rg^tm1Wjl^/SzJ* (NOD‐*scid IL2rγ^null^*, NSG) mice and NOD.Cg‐*Prkdc^scid^ Il2rg^tm1Wjl^* Tg(CMV‐IL3,CSF2,KITLG)1Eav/MloySzJ (NSG‐SGM3 mice) were obtained from The Jackson Laboratory (Bar Harbor, ME). All animals were housed in a specific pathogen free facility in microisolator cages, given autoclaved food and maintained on sulphamethoxazole‐trimethoprim medicated water (Goldline Laboratories, Ft Lauderdale, FL) and acidified autoclaved water on alternate weeks. All experiments were performed in accordance with the guidelines of the Institutional Animal Care and Use Committee of the University of Massachusetts Medical School and the recommendations in the Guide for the Care and Use of Laboratory Animals (Institute of Laboratory Animal Resources, National Research Council, National Academy of Sciences, 1996).

### Generation of BLT mice

Male and female NSG and NSG‐SGM3 mice at 6–10 weeks of age were irradiated with 100 cGy and implanted with human fetal thymus and liver fragments under the kidney capsule. The fetal tissues (gestational age 16–20 weeks) were obtained from Advanced Bioscience Resources (Alameda, CA). The tissues were washed with RPMI supplemented with penicillin G (100 U/ml), streptomycin (100 mg/ml), fungizone (0.25 μg/ml), and gentamycin (5 μg/ml) and 1 mm^3^ fragments of the fetal thymus and liver were implanted in the renal subcapsular space. Mice were injected subcutaneously with gentamycin (0.2 mg) and cefazolin (0.83 mg) post‐surgery. To obtain fetal HSC, fetal liver tissue was processed as described previously 15, depleted of CD3^+^ T cells and a cell suspension containing 1 to 2 × 10^5^ CD34^+^ fetal liver HSC was injected in the tail vein of mice between 4 and 6 h after irradiation.

### Antibodies and flow cytometry

Fluorophore‐linked primary antibodies (Supplemental Table S1) used for analysis of hematopoietic cell engraftment were purchased from BD Biosciences, Inc. (San Jose, CA), eBiosciences (San Diego, CA), or BioLegend (San Diego, CA). The following antibodies (clones) were used: mouse CD45 (30‐F11), human CD45 (2D1), CD34 (581), CD3 (UCHT1), CD20 (2H7), CD33 (WM53), CD4 (RPA‐T4), CD8 (RPA‐T8), CD25 (MA‐251 and 2A3), CD127 (A019D5), Foxp3 (236A/E7), CD45RA (HI100), CD27 (M‐T271), CD38 (HIT2), CD10 (HI10A), IgD (IAG‐2), CD138 (MI15). Single cell suspensions of spleen and bone marrow (recovered from one femur) were prepared from mice and whole blood was collected in heparin. Single cell suspensions of 0.5 to 1 × 10^6^ cells or 50–100 μl of heparinized whole blood were washed with FACS buffer (PBS with 2% FBS and 0.02% sodium azide) and incubated with rat anti‐mouse CD16/CD32 (clone 2.4G2) for 5–7 min at 4°C to block Fc binding. Cells were then incubated with antibodies for surface markers for 20 min at 4°C in the dark. Stained samples were washed with FACS buffer and fixed with 1% paraformaldehyde for cell suspensions or treated with BD FACS lysing solution for whole blood to lyse red blood cells (RBCs) and fix the samples. To detect human Tregs, blood samples were stained for surface markers, lysed and fixed and then incubated with eBioscience fixation/permeabilization buffer for 60 min. Cells were then stained with antibody against human Foxp3 in eBioscience permeabilization buffer for 60 min. At least 50,000 events were collected on LSRII flow cytometer (BD Biosciences, Inc, San Jose CA) using the BD FACSDIVA software. FlowJo software (Tree Star, Inc., Ashland, OR) was used to analyze data.

### Infections and ELISAs

Total human IgM and IgG concentrations were measured in the plasma of mice by ELISA as per the manufacturer's instructions (Bethyl Laboratories, Inc., Montgomery, TX) using an EMax Endpoint ELISA microplate reader (Molecular Devices, Sunnyvale, CA). To measure dengue virus specific antibody responses, mice were infected subcutaneously with approximately 10^6^ plaque forming units (PFUs) of dengue virus serotype‐2 strain New Guinea C (DENV‐2 NGC). The levels of dengue‐specific IgM and IgG were determined using inactivated dengue‐2 antigen lysates in ELISAs as described previously 12.

### Statistical analyses

Statistical analyses were performed using GraphPad PRISM software version 5 (GraphPad, San Diego, CA). An unpaired *t*‐test was performed to determine statistically significant differences between mean values of each data set.

## Results

### NSG‐SGM3 BLT mice show accelerated human cell chimerism as compared to NSG BLT mice

**Figure 1:**
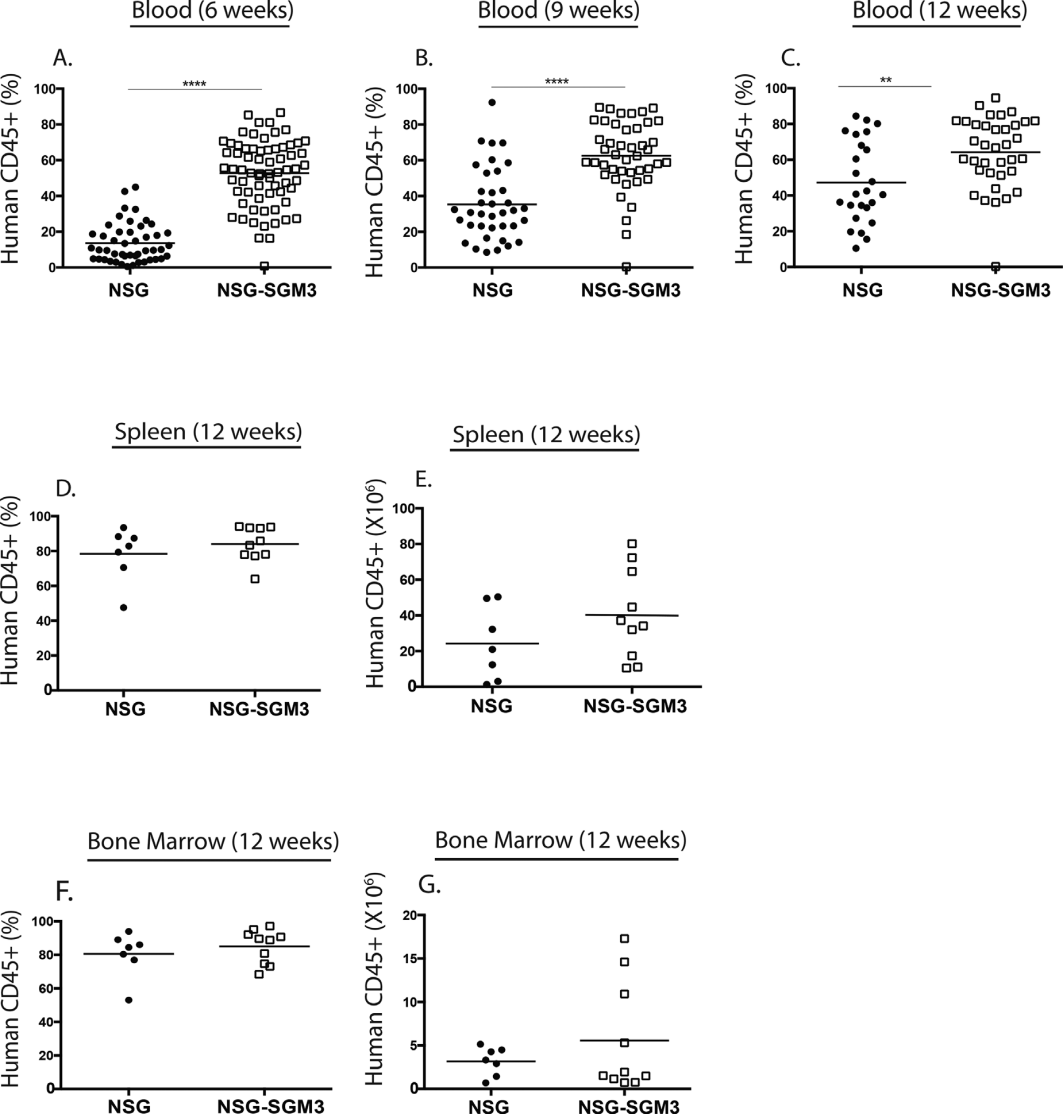
Human CD45^+^ cell engraftment kinetics in the peripheral blood, spleen, and bone marrow of NSG BLT mice and NSG‐SGM3 BLT mice. NSG and NSG‐SGM3 mice were irradiated with 100 cGy and implanted with 1 mm^3^ of human fetal thymus and liver in the renal subcapsular space. All mice were injected intravenously with 1 × 10^5^ CD34^+^ hematopoietic stem cells (HSC) derived from autologous fetal liver. The peripheral blood of the recipient NSG BLT and NSG‐SGM3 BLT mice was screened for total human hematopoietic CD45^+^ cell engraftment at the 6‐week (A), 9‐week (B), and 12‐week (C) post‐transplantation time points. The spleen (D and E) and bone marrow (F and G) of NSG BLT and NSG‐SGM3 BLT mice were screened for total human CD45^+^ cell engraftment 12 weeks after transplantation of human fetal tissues. Engraftment results are represented as a percentage of total cells or as total numbers in the spleen (D and E) and in the bone marrow (F and G). *p < 0.05; **p < 0.01; ****p < 0.0001. Each symbol indicates an individual BLT mouse. The results for peripheral blood are from four independent experiments and for spleen and bone marrow are from two independent experiments.

BLT mice were generated on the NSG or NSG‐SGM3 background and levels of human CD45^+^ hematopoietic cells were examined in the blood at 6, 9, and 12 weeks post‐implantation and in spleen and bone marrow at week 12 (Fig. 1). Levels of human CD45^+^ hematopoietic cells were significantly higher in the peripheral blood of NSG‐SGM3 mice at 6, 9, and 12 weeks as compared to NSG mice (Fig. 1A–C). The levels of circulating human CD45^+^ cells in NSG BLT mice continued to increase over time (13.7 ± 1.6% at 6 weeks, 35.3 ± 3.3% at 9 weeks, and 47.3 ± 4.6% at 12 weeks). In contrast, CD45^+^ cell levels in the blood of NSG‐SGM3 BLT mice reached maximal levels at 6 weeks and did not increase significantly beyond that time point (52.7 ± 2.2% at 6 weeks, 62.5 ± 2.9% at 9 weeks, and 64.2 ± 3.3% at 12 weeks). In the spleen, the percentages and total numbers of human CD45^+^ cells were similar between NSG and NSG‐SGM3 mice at 12 weeks post‐implantation (Fig. 1D and E). The percentages and total numbers of human CD45^+^ cells in the bone marrow were similar between NSG and NSG‐SGM3 mice at 12 weeks post‐implantation (Fig. 1F and G). Together these results indicated that NSG‐SGM3 mice support the rapid engraftment of human hematopoietic cells.

### NSG‐SGM3 BLT mice support human T cell development

**Figure 2:**
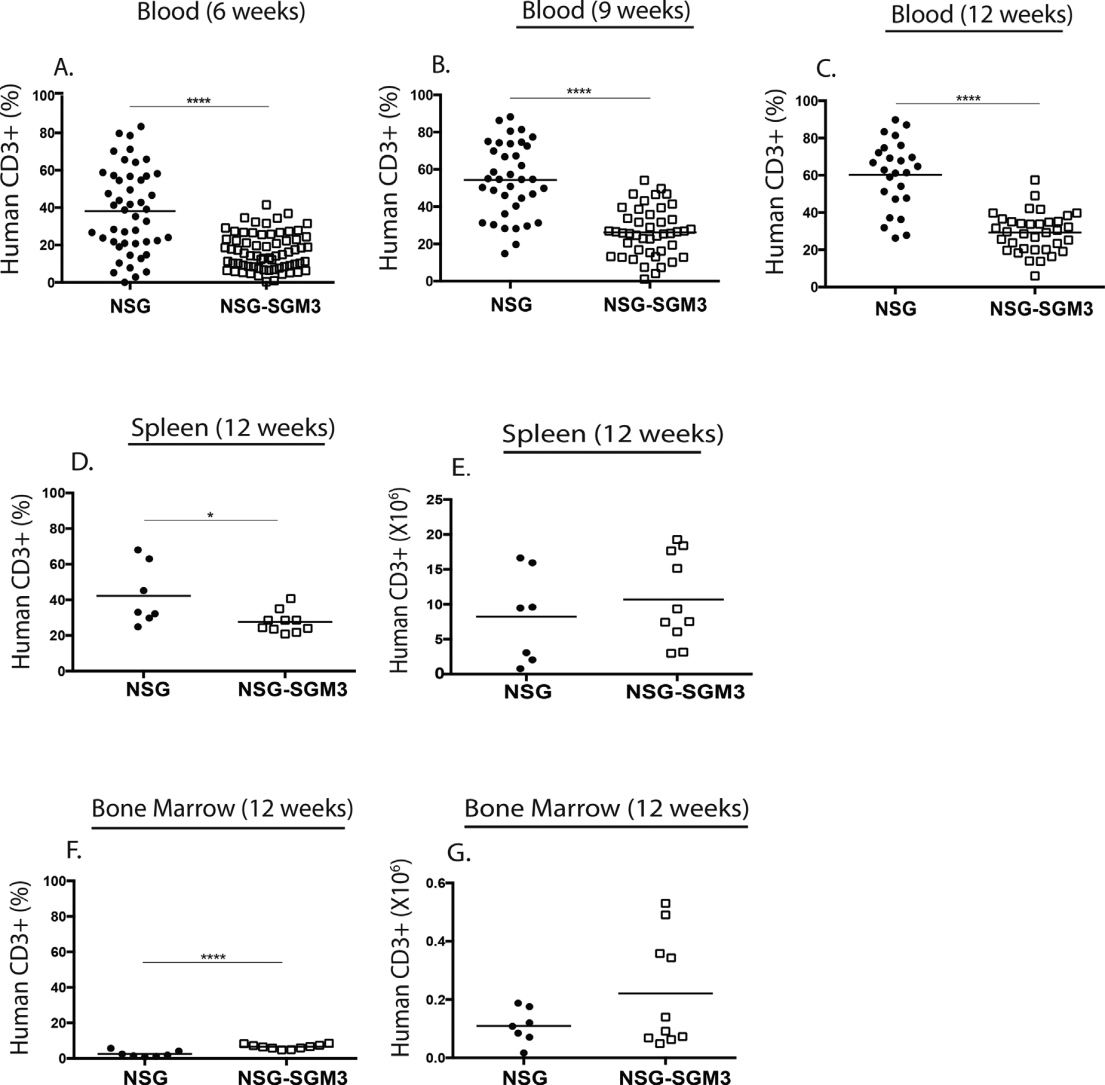
Human CD3^+^ T cell engraftment kinetics in the peripheral blood, spleen, and bone marrow of NSG BLT mice and NSG‐SGM3 BLT mice. The peripheral blood of the NSG BLT and NSG‐SGM3 BLT mice was screened for total human CD3^+^ T cell engraftment at 6‐week (A), 9‐week (B), and 12‐week (C) post‐transplantation of human fetal tissues. The spleen (D and E) and bone marrow (F and G) of NSG BLT and NSG‐SGM3 BLT mice were screened for human CD3^+^ T cell engraftment 12 weeks after transplantation of human fetal tissues. Engraftment results are represented as a percentage of total human CD45^+^ cells or as total numbers in the spleen (D and E) and in the bone marrow (F and G). Each symbol indicates an individual BLT mouse. The results for peripheral blood are from four independent experiments and for spleen and bone marrow are from two independent experiments.

A significant advantage of the BLT model is the efficient development of human T cells on autologous human thymic tissues. Human CD3^+^ T cell development in NSG‐SGM3 BLT and NSG BLT mice was monitored in the blood at 6, 9, and 12 weeks post‐implantation and in spleen and bone marrow at week 12 (Fig. 2). Levels of human T cells were significantly lower in the blood of NSG‐SGM3 mice compared to NSG mice and the differences were significant over time (Fig. 2A–C). T cell engraftment improved with the age of mice in both groups. NSG‐SGM3 mice had lower percentages of human T cells in the spleen compared to NSG mice at 12 weeks (Fig. 2D), but total numbers of T cells were similar (Fig. 2E). Human T cell levels were low in the bone marrow of both groups of mice, with significantly higher percentages (Fig. 2F) detected in NSG‐SGM3 mice compared to NSG mice and similar numbers for each group (Fig. 2G).

### NSG‐SGM3 BLT mice support human B cell development

**Figure 3:**
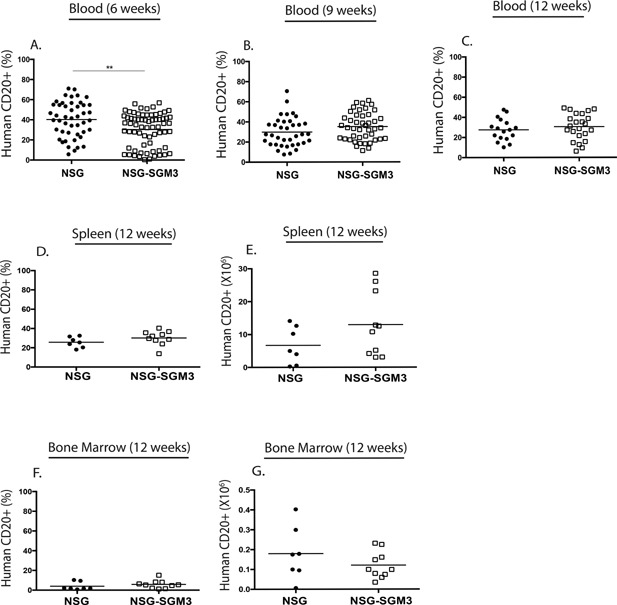
Human CD20^+^ B cell engraftment kinetics in the peripheral blood, spleen, and bone marrow of NSG BLT mice and NSG‐SGM3 BLT mice. The peripheral blood of the NSG BLT and NSG‐SGM3 BLT mice was screened for total human CD20^+^ B cell engraftment at 6‐week (A), 9‐week (B), and 12‐week (C) post‐transplantation of human fetal tissues. The spleen (D and E) and bone marrow (F and G) of NSG BLT and NSG‐SGM3 BLT mice were screened for human CD20^+^ B cell engraftment 12 weeks after transplantation of human fetal tissues. Engraftment results are represented as a percentage of total human CD45^+^ cells or as total numbers in the spleen (D and E) and in the bone marrow (F and G). *p < 0.05; **p < 0.01; ***p < 0.001; ****p < 0.0001. Each symbol indicates an individual BLT mouse. The results for peripheral blood are from four independent experiments and for spleen and bone marrow are from two independent experiments.

Human CD20^+^ B cell development in NSG‐SGM3 BLT and NSG BLT mice was monitored in the blood at 6, 9, and 12 weeks post‐implantation and in spleen and bone marrow at week 12 (Fig. 3). Levels of human B cells were significantly lower in the blood of NSG‐SGM3 mice at week 6 compared to NSG mice but the levels were comparable at weeks 9 and 12 (Fig. 3A–C). NSG‐SGM3 and NSG mice had similar percentages and total numbers of human B cells in the spleen (Fig. 3D and E) and bone marrow (Fig. 3F and G) at 12 weeks.

### NSG‐SGM3 BLT mice support enhanced myeloid cell development compared to NSG BLT mice

**Figure 4:**
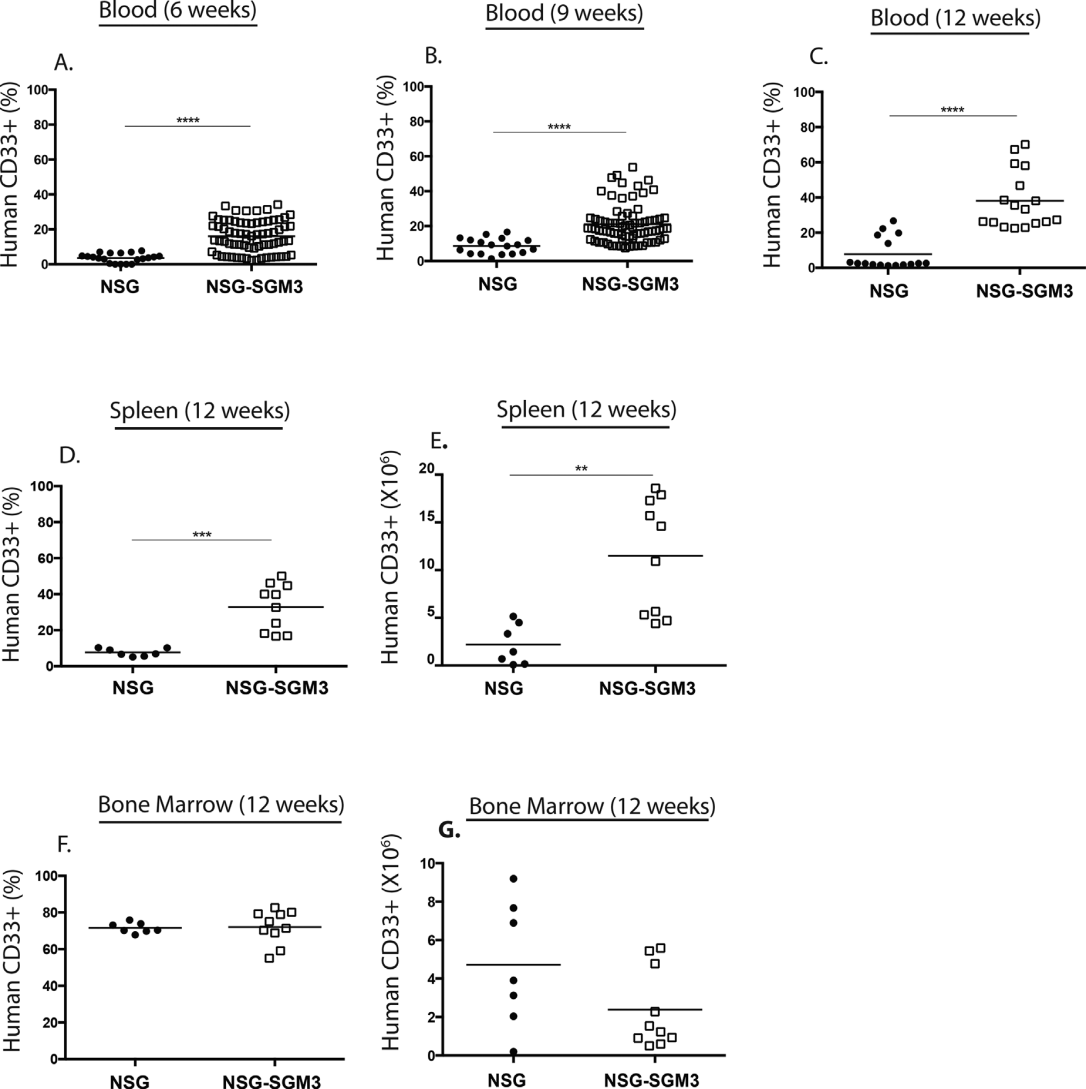
Human CD33^+^ myeloid cell engraftment kinetics in the peripheral blood, spleen, and bone marrow of NSG BLT mice and NSG‐SGM3 BLT mice. The peripheral blood of the NSG BLT and NSG‐SGM3 BLT mice was screened for total human CD33^+^ myeloid cell engraftment at 6‐week (A), 9‐week (B), and 12‐week (C) post‐transplantation of human fetal tissues. The spleen (D and E) and bone marrow (F and G) of NSG BLT and NSG‐SGM3 BLT mice were screened for human CD33^+^ myeloid cell engraftment 12 weeks after transplantation of human fetal tissues. Engraftment results are represented as a percentage of total human CD45^+^ cells or as total numbers in the spleen (D and E) and in the bone marrow (F and G). In (B), data points shown for NSG‐SGM3 BLT are a combination of 8 and 9 week time points. *p < 0.05; **p < 0.01; ***p < 0.001; ****p < 0.0001. Each symbol indicates an individual BLT mouse. The results for peripheral blood are from four independent experiments and for spleen and bone marrow are from two independent experiments.

Previous studies have shown that NSG‐SGM3 mice engrafted with human HSC have significantly improved myeloid cell development 18, 19, 20, 21. Human CD33^+^ myeloid cell development in NSG‐SGM3 BLT and NSG BLT mice was monitored in the blood at 6, 9, and 12 weeks post‐implantation and in spleen and bone marrow at week 12 (Fig. 4). At all time points tested significantly higher levels of human CD33^+^ cells were detected in the blood of NSG‐SGM3 BLT mice as compared to NSG BLT mice (Fig. 4A–C). In the spleen, the percentages and total numbers of human CD33^+^ cells were significantly higher in NSG‐SGM3 mice at 12 weeks post‐implantation compared to NSG mice (Fig. 4D and E). The percentages and total numbers of human CD45^+^ cells in the bone marrow were similar between NSG and NSG‐SGM3 mice at 12 weeks (Fig. 4F and G). Together, these data show that NSG‐SGM3 BLT mice show a heightened development of human myeloid development as has been found in HSC‐engrafted NSG‐SGM3 mice 21.

### NSG‐SGM3 BLT mice show improved engraftment of CD4^+^ regulatory T cells as compared to NSG BLT mice

**Figure 5:**
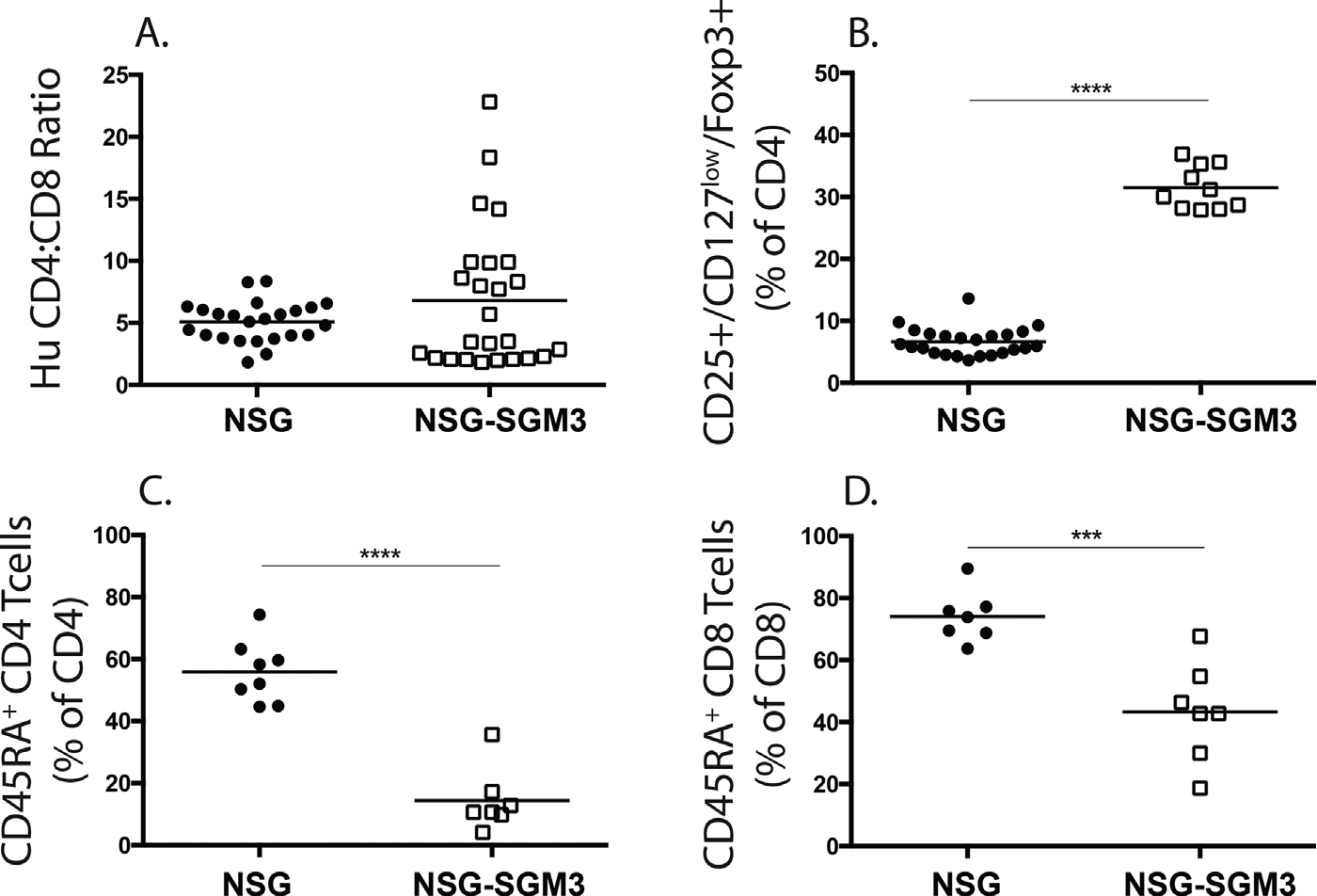
Characterization of human CD3^+^ T cells in the peripheral blood of NSG BLT and NSG‐SGM3 BLT mice at 12 week‐post transplantation. The ratio of human CD4:CD8 T cells gated on CD3^+^ T cells is shown in (A). The proportion of CD25^+^CD127^low^Foxp3^+^ regulatory T cells (Treg) gated on CD4^+^ T cells is shown in (B). The percentages of CD4^+^ and CD8^+^ T cells expressing naïve T cell marker CD45RA are shown in (C) and (D), respectively. ***p < 0.001; ****p < 0.0001. Each symbol indicates an individual BLT mouse. The results for peripheral blood are from two independent experiments.

NSG‐SGM3 mice engrafted with human HSC have previously been shown to have enhanced development of CD4^+^ human Tregs 21. We characterized T‐cell subsets in NSG‐SGM3 BLT mice, by comparing CD4^+^ to CD8^+^ T cell ratios, Treg levels and T cell phenotype in the blood of both groups of mice at 12 weeks post‐tissue implantation (Fig. 5). The CD4^+^ to CD8^+^ ratio was approximately 5:1 in both strains of mice (Fig. 5A). Significantly higher levels of human CD4^+^CD25^+^CD127^lo^Foxp3^+^ T regulatory cells were detected in the blood of NSG‐SGM3 mice compared to NSG mice (Fig. 5B). Analysis of CD45RA expression levels by human CD4 and CD8 T cells showed a lower proportion of T cells expressing CD45RA in the blood of NSG‐SGM3 mice compared to NSG mice (Fig. 5C and D). These data indicate that NSG‐SGM3 BLT mice have higher levels of CD4^+^ regulatory T cells, and lower levels of naïve CD4^+^ and CD8^+^ T cells.

### NSG‐SGM3 BLT mice develop higher levels of mature naïve B cells compared to NSG BLT mice

**Figure 6:**
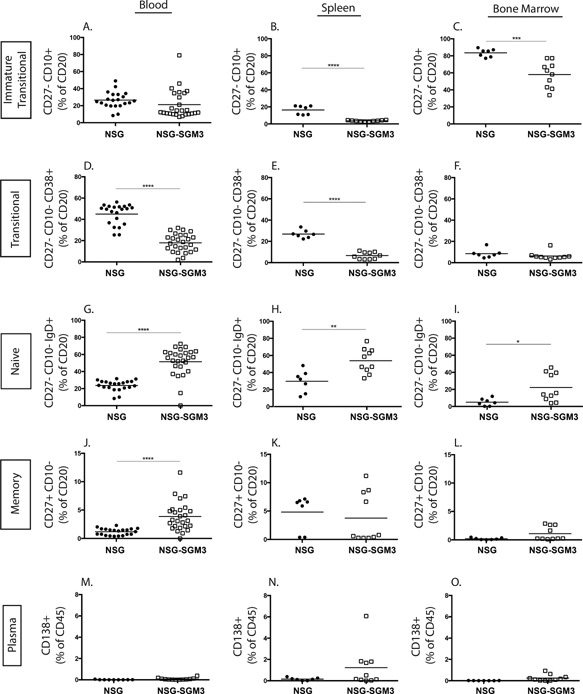
Characterization of human CD20^+^ B cells in the peripheral blood, spleen, and bone marrow of NSG BLT and NSG‐SGM3 BLT mice at 12 weeks post‐transplantation. Human B cells were divided into five categories and expressed as a percentage of total human CD20^+^ B cells: CD20^+^CD27^−^CD10^+^ immature transitional B cells in blood (A), spleen (B), bone marrow (C); CD20^+^CD27^−^CD10^−^CD38^+^ transitional B cells in blood (D), spleen (E), bone marrow (F); CD20^+^CD27^−^CD10^−^IgD^+^ mature naïve B cells in blood (G), spleen (H), bone marrow (I); CD20^+^CD27^+^CD10^−^ memory B cells in blood (J), spleen (K), bone marrow (L) and CD20^+^CD138^+^ plasma cells in blood (M), spleen (N), bone marrow (O). *p < 0.05; **p < 0.01; ***p < 0.001; ****p < 0.0001. Each symbol indicates an individual BLT mouse. The results for peripheral blood are from three independent experiments and for spleen and bone marrow are from two independent experiments.

Previous studies have described a predominance of immature B cells in humanized mice 11, 14, 15. To evaluate B cell development in NSG‐SGM3 BLT mice, we examined the phenotype of B cells in the periphery (Fig. 6). B cells were categorized into five groups based on their phenotypic markers; immature/transitional B cells (CD27^−^CD10^+^), transitional B cells (CD27^−^CD10^−^CD38^+^), mature naïve B cells (CD27^−^CD10^−^IgD^+^), memory B cells (CD27^+^CD10^−^), and plasma cells (CD138^+^) (for gating strategy see Supplemental Fig. S1). Immature/transitional B cells were present at comparable levels in the peripheral blood of NSG‐SGM3 and NSG mice (Fig. 6A). NSG‐SGM3 mice had lower levels of immature/transitional B cells in the spleen (Fig. 6B) and bone marrow (Fig. 6C). Transitional human B cells were significantly lower in blood (Fig. 6D) and spleen (Fig. 6E) of NSG‐SGM3 mice compared to NSG mice and levels were similar in the bone marrow (Fig. 6F). Mature/naïve B cells were significantly higher in the blood (Fig. 6G), spleen (Fig. 6H) and bone marrow (Fig. 6I) of NSG‐SGM3 mice compared to control NSG mice. Healthy human adults have between 60–70% mature naïve B cells in the peripheral blood 22. Memory B cells were present at significantly higher levels in the blood of NSG‐SGM3 mice compared to NSG mice (Fig. 6J) and similar in spleen and bone marrow (Fig. 6K and L). Healthy human adults are reported to have 10–30% memory B cells circulating in blood 22. Plasma cells were present at very low levels in both groups of mice in all tissues (Fig. 6M–O). Together these data indicate that NSG‐SGM3 BLT mice have lower levels of immature and transitional B cells and higher levels of mature naïve B cells relative to NSG BLT mice, suggesting that the NSG‐SGM3 BLT mice have improved human B cell maturation.

### NSG‐SGM3 BLT mice show an improved ability to generate IgG antibodies

**Figure 7:**
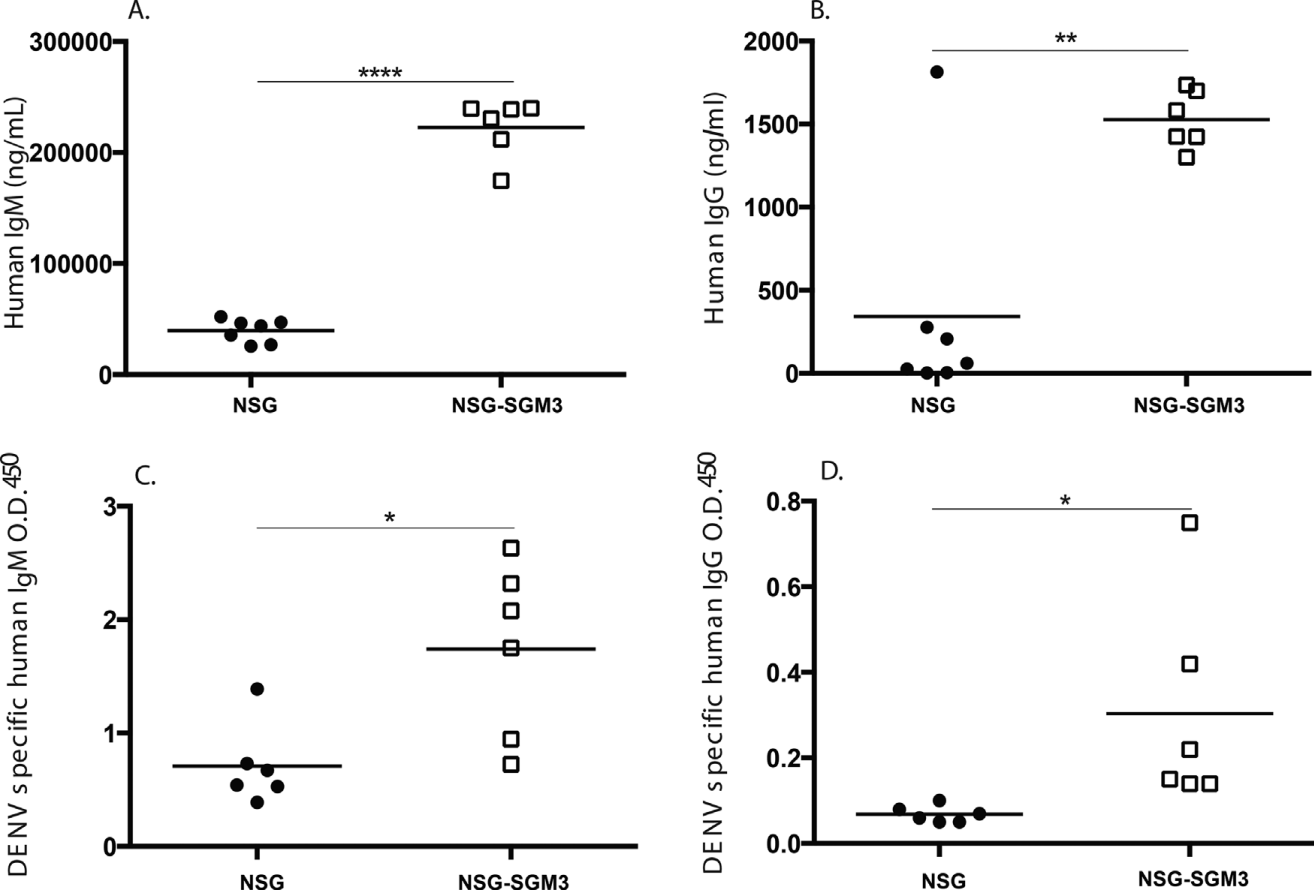
Evaluation of total antibody titers and dengue virus specific antibody responses in NSG BLT and NSG‐SGM3 BLT mice. Mice were bled at 12 weeks post‐implantation of human tissues and total human IgM (A) and human IgG (B) levels (ng/ml) were determined in the plasma of these mice by ELISA. Mice were infected with DENV‐2 and plasma samples were tested to determine DENV‐2 specific IgM (C) and IgG (D) by sandwich ELISA. Each symbol indicates an individual BLT mouse. The results from two independent experiments.

A major limitation of humanized mice is their reduced ability to generate human IgG responses. The enhanced human B cell maturation observed in the NSG‐SGM3 BLT mouse model (Fig. 6) suggested that these mice may have an increased ability to undergo Ig class switching. The basal levels of human IgM and IgG in the plasma of resting BLT mice were therefore evaluated at 12 weeks post‐tissue implant. NSG‐SGM3 mice had 5.6‐fold higher levels of human IgM compared to NSG mice (Fig. 7A). Human IgG levels were 4.5‐fold higher in NSG‐SGM3 mice compared to NSG mice (Fig. 7B). Next we infected the mice with DENV‐2 (dengue virus serotype‐2) and assessed the generation of DENV‐2 specific antibodies by sandwich ELISA 4 weeks post‐infection. We previously demonstrated the generation of IgM responses to the inactivated lysates of dengue antigen and the DENV‐2 E (envelope) protein in NSG BLT mice but limited antigen‐specific IgG responses 23. NSG‐SGM3 BLT mice infected with DENV‐2 had significantly higher levels of DENV‐2 specific IgM (Fig. 7C) and increased levels of DENV‐2 specific IgG (Fig. 7D). These data indicate that transgenic expression of SCF, GM‐CSF, and IL‐3 generates higher levels of total human IgM and IgG suggesting improved class switching and induces improved viral antigen‐specific antibody responses.

## Discussion

The BLT model enables the generation of humanized mice having a complete human immune system, including the development of both conventional and regulatory HLA‐restricted T cells. Although B cells develop in BLT mice, the circulating antibody levels are significantly lower compared to adult humans. Moreover, the generation of antigen‐specific antibody responses in BLT mice, specifically IgG responses, are weak, limiting their use in studying humoral responses to pathogens and testing candidate vaccines 10. Two studies have demonstrated that the immunoglobulin gene repertoires of human B cells in humanized (HSC‐engrafted) mice are similar to those of normal human peripheral B cells suggesting that humanized mice have the genetic potential to mount broad and high affinity antibody responses to diverse pathogens 24, 25. The poor antigen‐specific antibody responses are unlikely to be attributed to a genetic defect in the immunoglobulin repertoire. Studies have suggested that the contributing factors are defects in T and B cell maturation, disorganized secondary lymphoid structures, and an absence of human cytokines 13, 14, 15, 16, 17.

Mature B cells that encounter their cognate antigen in lymphoid follicles and receive T cell help enter a germinal center where they undergo the processes of class switching, that generates different antibody isotypes, and somatic hypermutation, that diversifies their antibody repertoire 26. Activated B cells can then differentiate into plasmocytes and become antibody‐secreting plasma cells or become long‐lived memory B cells. Human B cells develop in NSG‐BLT mice and generate human immunoglobulins although at lower levels than in adult humans 15. Human B cells in humanized mice have been shown to generate antigen‐specific antibody responses to human immunodeficiency virus 1 (HIV‐1) 11, 27, West Nile virus 11, dengue virus 23, and Epstein–Barr virus (EBV) 28. These antibody responses are predominantly of the IgM isotype with very little to no IgG responses. A few studies have demonstrated antigen‐specific IgG responses in a small subset of mice infected with HIV‐1 29, 30, albeit at low titers. The inability to efficiently generate antibody response to most antigens has necessitated the development of humanized mouse models with improved class‐switching.

The expression of human transgenes and inactivation of specific mouse genes has improved humoral responses in some models. Transgenic expression of human signal regulatory protein alpha (SIRP alpha) in BALB/c‐*Rag2^−/−^ IL2rg^null^* mice that were injected with human HSC significantly elevated the total levels of human IgG in the plasma, but could not elicit a strong IgG response to the protein antigen ovalbumin (OVA) 31. However, transgenic expression of HLA‐DR4 in NOD‐*Rag1^−/−^ IL2rg^null^* mice engrafted with HLA‐DR4^+^ HSC elicited an IgG response to tetanus toxoid vaccine as well as class switching to IgA, IgE, and all subtypes of IgG 32. In addition HLA‐DR4 expression in NOD *Shi‐scid IL2rg^null^* mice induced an anti‐OVA IgG response 33. Expression of specific human cytokines has also improved the engraftment of human hematopoietic cells and enhanced the development and function of human immune cells, including B cells 16. Administration of the recombinant human cytokine BAFF (B cell activating factor, also called BLyS) to NOD‐*Rag1^−/−^ Prf1^−/−^* mice injected with human peripheral blood lymphocytes (PBL) improved the engraftment of B cells, elevated serum immunoglobulin levels, and generated an antibody response to thymus‐independent antigens in pneumovax vaccine 34. Hydrodynamic injection of human GM‐CSF and IL‐4 in HSC‐engrafted NSG mice allowed the induction of tetanus toxoid specific IgG as well as neutralizing antibodies against H5N1 influenza virus upon immunization 35. While the approaches described above have improved human B cell functionality in humanized mice, further advancements are necessary to make these models applicable to study human B cell immunity.

In this study, we have shown that NSG‐SGM3 BLT mice that transgenically express human SCF (also called c‐kit ligand or Steel factor), GM‐CSF and IL‐3 show heightened human B cell engraftment consistent with the important role of these human hematopoietic growth factors in hematopoiesis as well as in proliferation and survival of HSC in vitro 36. Moreover, NSG‐SGM3 mice show accelerated kinetics of human immune system development as compared to NSG‐BLT mice (Fig. 1). In our initial studies that have followed survival of NSG‐BLT and NSG‐SGM3 BLT mice, we have noted earlier onset of GVHD‐symptoms in the SGM3 mice, generally occurring 3 weeks prior to NSG mice (data not shown). Given these accelerated kinetics, NSG‐SGM3 mice should be used in experiments beginning at 9 weeks post‐engraftment. Three studies have described HSC‐engrafted NSG mice transgenically expressing membrane bound SCF 20, 37, 38. In one study, transgenic expression of membrane bound SCF circumvented the need for irradiation and permitted high levels of engraftment of human CD45^+^ cells in HSC engrafted NSG mice 37. Another study reported these mice to have an improved human myeloid cell compartment, specifically cells of the granulocytic lineage 38. A recent study showed that the human myeloid cells in the SCF NSG mice migrated to the renal tissue to become resident dendritic cells and some of these mice could be used as a source of human bone marrow‐derived macrophages 20. GM‐CSF and IL‐3 are important for the development and function of myeloid cells. Human IL‐3/GM‐CSF knock‐in mice engrafted with human HSC were shown to have improved myeloid cell reconstitution in the lung and the engrafted human alveolar macrophages mounted an innate immune response to influenza virus showing that the myeloid cells were functionally responsive 39. NSG‐SGM3 mice engrafted with human HSC (the Hu‐SRC‐SCID model) have been described previously 21. These mice were shown to have elevated myeloid cell frequencies, specifically myeloid DCs, and CD4^+^ Foxp3^+^ regulatory T cells. NSG‐SGM3 BLT mice have a similar increase in myeloid cell levels (Fig. 4) and Tregs (Fig. 5B).

Interestingly, NSG‐SGM3 BLT mice had significantly higher frequencies of mature naïve B cells and proportionately reduced frequencies of immature and transitional B cells compared to control NSG BLT mice (Fig. 6). Immature B cells develop from the lymphoid progenitors in the bone marrow by passing through the pro‐B and pre‐B cell stages. These immature B cells expressing surface IgM (sIgM) exit the bone marrow and enter into the periphery. The early bone marrow emigrants are called immature transitional B cells and they express the markers CD10 and CD38 40. In the periphery, they develop into transitional B cells that can gain access to lymphoid follicles in the spleen and become more sensitive to T cell help. Upon receiving appropriate cytokine signals and positive signals through the B‐cell receptor, they become mature B cells that repopulate the periphery 41. Successful differentiation from the transitional to mature B cell stage is governed by the cytokine BAFF 42. BAFF is a member of the TNF (tumor necrosis factor) family and is secreted mainly by myeloid cells such as neutrophils, monocytes, macrophages, and dendritic cells 43. BAFF is also shown to be produced by activated T cells to some extent 44. We propose two explanations for improved B cell maturation observed in NSG‐SGM3 BLT mice. Firstly, the increased frequencies of activated T cells characterized as CD45RA^−^ (Fig. 5C and D) could facilitate B cell maturation in NSG‐SGM3 mice. Recently, Lang et al. demonstrated a requirement of T cells for human B cell maturation in BALB/c‐*Rag2^−/−^ IL2rg^null^* mice engrafted with CD34^+^ HSC 45. The study showed that adoptive transfer of autologous T cells elevated mature B cell frequencies, whereas T cell depletion diminished mature B cell levels. T cell activation, characterized by the expression of CD45RO, HLA‐DR and CD49d, correlated with B cell maturation suggesting T cell activation might be important for B cell maturation. We did not find increased numbers of T cells in NSG‐SGM3 BLT mice relative to NSG BLT mice (Fig. 2). However, the increased frequencies of CD45RA^−^ activated T cells (Fig. 5C and D) in NSG‐SGM3 BLT mice could possibly explain the improved B cell maturation in these mice. Secondly, the well engrafted human myeloid cell compartment in the NSG‐SGM3 mice (Fig. 4) could secrete increasing amounts of human BAFF that binds the BAFF‐R on transitional B cells rescuing them from death and easing them into the mature B cell stage. Also consistent with its role of a “survival cytokine,” BAFF would bind BAFF‐R on mature B cells mediating their longevity and thus elevating the mature B cell frequencies. Currently, we are evaluating the role of BAFF in driving B cell maturation in NSG‐SGM3 BLT mice.

Since improved B cell maturation contributes to enhanced B cell functionality in terms of antigen‐specific antibody responses, we tested the hypothesis that improved B cell maturation in NSG‐SGM3 BLT mice would generate antigen‐specific antibody responses. Previously, we demonstrated that NSG BLT mice can elicit DENV‐2 antigen‐specific antibody responses which are predominantly of the IgM class 23. NSG‐SGM3 BLT mice elicited significantly stronger IgM as well as IgG responses to inactivated DENV‐2 lysates relative to NSG BLT mice. We propose that improved B cell maturation in these mice contributes to the enhanced levels of human immunoglobulins and antigen‐specific antibody responses. The histology sections of spleens of NSG and NSG‐SGM3 BLT mice upon dengue infection showed similarly disorganized architecture ruling out the formation of organized secondary lymphoid structures in NSG‐SGM3 mice (data not shown). However, we cannot rule out other mechanisms for enhanced antibody responses in these mice.

To summarize, transgenic expression of SCF, GM‐CSF, and IL‐3 in NSG BLT mice resulted in improved human myeloid cell reconstitution, enhanced B cell maturation, and antigen‐specific antibody responses to dengue virus infection. Thus, NSG‐SGM3 BLT mice prove to be a useful tool to study antibody responses to viral infections. Further improvements in B cell development, such as the development of secondary lymphoid structures to support class switching, engraftment of human follicular dendritic cells, and enhanced B and T cell maturation, are necessary to achieve strong humoral responses. In the future, supplementation of humanized mice with human “survival cytokines” like BAFF and IL‐7 might be a positive step in resolving the issue of impaired B cell development and function.

## Author contributions

SJ and AM performed experiments. MB and LS designed experiments. SJ, MB, and LS wrote the manuscript.

## Conflict of Interest

MB is a consultant for the Jackson Laboratory.

## Supporting information

Additional supporting information may be found in the online version of this article at the publisher's web‐site.

**Figure S1**. Gating strategy to identify human B cell subsets.

**Table S1**. Antibodies used for flow cytometry.
