# Supplementary material for: Improved B cell development in humanized NOD‐scid IL2Rγnull mice transgenically expressing human stem cell factor, granulocyte‐macrophage colony‐stimulating factor and interleukin‐3
Source: Immun Inflamm Dis. 2016 Aug 28;4(4):427–40. doi: 10.1002/iid3.124 (PMC5134721; doi:10.1002/iid3.124)
Supplement: Supplementary file 1 — Figure S1. Gating strategy to identify human B cell subsets. [file IID3-4-427-s001.pdf]

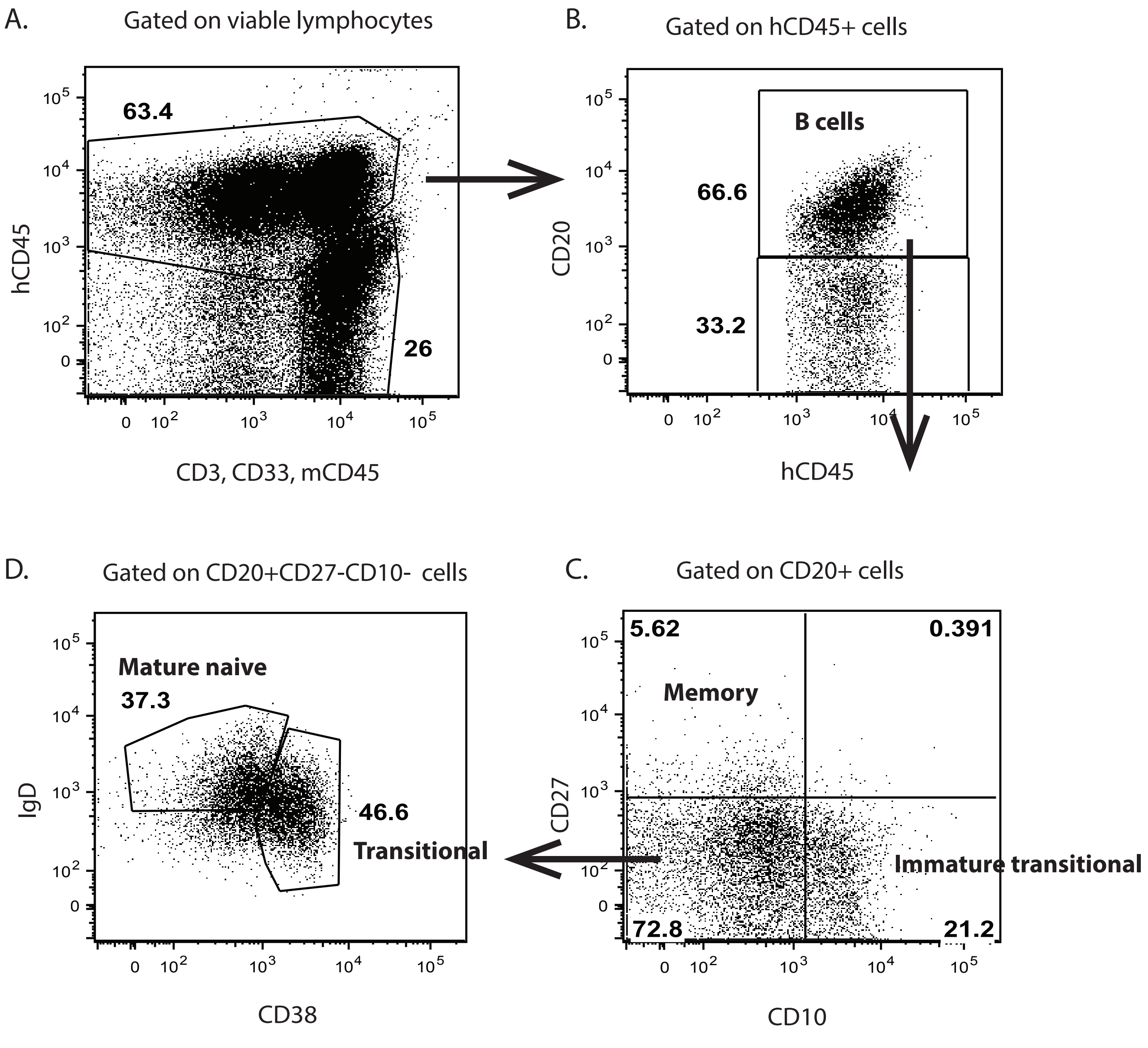

Supplemental Figure 1. Gating strategy to identify human B cell subsets. 1A shows human CD45+ cells gated on viable lymphocytes. 1B shows human CD20+ cells gated on human CD45+ cells. 1C shows CD20+CD27+CD10- memory B cells and CD20+CD27-CD10+ transitional B cells. 1D shows CD20+CD27-CD10-IgD+ mature naïve B cells and CD20+CD27-CD10-CD38+ immature transitional B cells.
