# Supplementary material for: Improved B cell development in humanized NOD‐scid IL2Rγnull mice transgenically expressing human stem cell factor, granulocyte‐macrophage colony‐stimulating factor and interleukin‐3
Source: Immun Inflamm Dis. 2016 Aug 28;4(4):427–40. doi: 10.1002/iid3.124 (PMC5134721; doi:10.1002/iid3.124)
Supplement: Supplementary file 2 — Table S1. Antibodies used for flow cytometry. [file IID3-4-427-s002.docx]

| Supporting Table 1. Antibodies used for flow cytometry | | | |
| --- | --- | --- | --- |
| Marker | **Clone** | **Fluorophore** | **Antibody Dilution** |
| Mouse CD45 | 30-F11 | PerCP-Cy5.5 | 1:200 |
| Human CD45 | 2D1 | APC-H7 | 1:200 |
| Human CD34 | 581 | PE | 1:50 |
| Human CD3 | UCHT1 | PerCP-Cy5.5, FITC | 1:100 |
| Human CD20 | 2H7 | APC-H7 | 1:100 |
| Human CD33 | WM53 | PerCP-Cy5.5, FITC | 1:100 |
| Human CD4 | RPA-T4 | Alexa Fluor 700 | 1:100 |
| Human CD8 | RPA-T8 | Pacific Blue | 1:100 |
| Human CD25 | MA-251 | APC | 1:40 |
| Human CD25 | 2A3 | APC | 1:10 |
| Human CD127 | A019D5 | PE-Cy7 | 1:100 |
| Human Foxp3 | 236A/E7 | PE | 1:20 |
| Human CD45RA | HI100 | APC | 1:50 |
| Human CD27 | M-T271 | AmCyan | 1:100 |
| Human CD38 | HIT2 | PE | 1:20 |
| Human CD10 | HI10A | PE-Cy7 | 1:20 |
| Human IgD | IAG-2 | FITC | 1:100 |
| Human CD138 | MI15 | Pacific Blue | 1:20 |
